# Structural and functional studies of ferredoxin and oxygenase components of 3-nitrotoluene dioxygenase from *Diaphorobacter* sp. strain DS2

**DOI:** 10.1371/journal.pone.0176398

**Published:** 2017-04-27

**Authors:** Archana Kumari, Deepak Singh, S. Ramaswamy, Gurunath Ramanathan

**Affiliations:** 1 Department of Chemistry, Indian Institute of Technology Kanpur, Kalyanpur, Kanpur, Uttar Pradesh, India; 2 Institute for Stem Cell Biology and Regenerative Medicine, National Center for Biological Science, Tata Institute of Fundamental Research, Bangalore, Karnataka, India; Russian Academy of Medical Sciences, RUSSIAN FEDERATION

## Abstract

3-nitrotoluene dioxygenase (3NTDO) from *Diaphorobacter* sp. strain DS2 catalyses the conversion of 3-nitrotoluene (3NT) into a mixture of 3- and 4-methylcatechols with release of nitrite. We report here, X-ray crystal structures of oxygenase and ferredoxin components of 3NTDO at 2.9 Å and 2.4 Å, respectively. The residues responsible for nitrite release in 3NTDO were further probed by four single and two double mutations in the catalytic site of α-subunit of the dioxygenase. Modification of Val 350 to Phe, Ile 204 to Ala, and Asn258 to Val by site directed mutagenesis resulted in inactive enzymes revealing the importance of these residues in catalysis. Docking studies of *meta* nitrotoluene to the active site of 3NTDO suggested possible orientations of binding that favor the formation of 3-methylcatechol (3MC) over 4-methylcatechol energetically. The electron transfer pathway from ferredoxin subunit to the active site of the oxygenase subunit is also proposed.

## Introduction

Nitroaromatic compounds are widely used in the production of dyes, pigments, pesticides and explosives [1]. These compounds are toxic and recalcitrant due to the inherent stability of the nitroaromatic ring. Bacterial strains that completely mineralize nitrobenzene [2], 2-nitrotoluene (2NT)[3], 3-nitrotoluene (3NT)[4], 2,4-dinitrotoluene, 2,6-dinitrotoluene have been isolated and characterized previously[5, 6]. *Diaphorobacter* sp. strain DS2 utilizes 3-nitrotoluene as a sole source of carbon, nitrogen and energy [4]. The initial enzyme in the degradation pathway is 3-nitrotoluene dioxygenase (3NTDO) which catalyses the oxidation of 3-nitrotoluene into a mixture of methylcatechols and nitrite [7]. 3NTDO is a multicomponent enzyme system which adds two atoms of molecular oxygen to the substrate through the sequential action of three separate proteins[7]. In this reaction, electrons originating from NADH/NADPH are transferred to FAD in the reductase, the first redox center which subsequently reduces the plant type [2Fe-2S] cluster in the reductase that, in turn, reduces the terminal oxygenase through a ferredoxin. The terminal oxygenase is an α_3_β_3_ hetero-hexamer wherein the catalytic α subunits contain a Rieske [2Fe-2S] cluster and mononuclear iron at the active site[8]. The catalytic mononuclear iron is coordinated by two histidines and an aspartic acid. This *His*-*His*-carboxylate facial triad is conserved in all known Rieske oxygenases (ROs) [9]. Naphthalene dioxygenase (NDO) from *Pseudomonas* sp. strain NCIB9816-4 is the first dioxygenase for which a high resolution crystal structure has been reported[10].

3NTDO from *Diaphorobacter* sp. strain DS2 belongs to the family of naphthalene dioxygenases, and has the ability to remove nitrite from all isomers of mononitrotoluene, 2-chloronitrobenzene, 2,6-dinitrotoluene and partially from 3-chloronitrobenzene[11, 12]. Active site residues of 3NTDO were compared with other nitroarene dioxygenases like nitrobenzene dioxygenase (NBDO) from *Comamonas* sp. JS765[13], 2-nitrotoluene dioxygenase (2NTDO) from *Acidovorax* sp. strain JS42[14], 2,4-dinitrotoluene dioxygenase (DNTDO) from *Burkholderia* sp. strain DNT[15]and *Burkholderia cepacia* R34[16]. It has been reported that Asn258 is an important residue for nitrite removal in NBDO[17] and 2NTDO[18]. Crystal structure of NBDO bound with 3NT shows the hydrogen bonding between nitro group of the substrate with Asn258[13]. Similarly, DNTDO removes one nitrite from 2,4-dinitrotoluene (2,4-DNT) producing a catechol and has a valine at 258 instead of aspargine in its active site[19]. This system is also known to give 4-methyl catechol (4MC) from 4NT but is unable to remove nitrite from 2NT and 3NT[20]. NDO variants from *Ralstonia* U2 at Phe350 and Gly407 positions yield 3-methyl-4-nitrocatechol from 2,6-DNT with the removal of one nitrite group and *Ralstonia* U2 does not have Asn258[21]. However, NDO variants from *Pseudomonas putida* NCIB9816-4 have been reported to be unable to detach nitrite from any nitroaromatic compound even when Val 260 was modified to Asn[22]. In order to elucidate the atomic resolution details of the unique specificity of 3NTDO, we solved the crystal structure of the oxygenase component of 3NTDO and selected four active site residues for site directed mutagenesis. The docking of 3NT to the oxygenase structure has also been carried out. Here, we also report the crystal structure of the ferredoxin component of 3NTDO and we propose the residues involved in the electron transfer pathway from ferredoxin to the mononuclear iron center at the active site of the oxygenase for naphthalene dioxygenase family members.

## Materials and methods

### Bacterial strains, plasmids and growth conditions

The reductase, ferredoxin and oxygenase components of 3NTDO from *Diaphorobacter* sp. strain DS2 were cloned separately in pET21a vector and reported previously[11]. *E*.*coli* BL21(DE3)(pDS5), BL21(DE3)(pDS2) and BL21(DE3)(pDS21) were grown in minimal salts medium supplemented with 10mM glucose, 1mM thiamine and 200μg/ml ampicillin for the reductase, ferredoxin and oxygenase, respectively [23]. Cells were harvested by centrifugation and pDS21 cells were resuspended in BTGD buffer [50mM bis-tris (pH 6.8), 5% (v/v) glycerol, 1mM dithiothreitol] and pDS5 and pDS2 cells were suspended in MGD buffer [25mM MOPS (pH 7.4), 5% glycerol, 1mM DTT] with 50 and 100mM NaCl, respectively and frozen at -70°C.

### Purification of reductase

Cell extracts(BL21(DE3)(pDS5))were applied to a HiPrep QFF(20ml) column equilibrated with 25mM 3-(N-morpholino) propanesulfonic acid (MOPS), 50mM NaCl, 5% glycerol and 1mM DTT(buffer 1). Unbound proteins were eluted with six column volumes of this buffer and the bound protein was eluted using a linear gradient of 0.05M to 0.8M NaCl. The reductase fractions were pooled and concentrated using amicon^®^ MWCO30 kDa filter. Ammonium sulphate was added to a final concentration of 1M, incubated for 2h and centrifuged for 20min at 20,000g. The supernatant was loaded onto a Hitrap phenyl sepharose HP column equilibrated with a buffer I containing 1M ammonium sulphate. Unbound protein was eluted with a buffer containing ammonium sulphate and the bound protein was eluted at linear gradient from 1M to 0M ammonium sulphate. The reductase fractions were pooled and concentrated and finally loaded onto Hiload superdexS-75 gel filtration column equilibrated with buffer 1. A monodispersed peak of the reductase was observed. The peak fractions were pooled, concentrated and buffer exchanged with 50mM MES buffer, pH 6.8 and finally stored at -80°C.

### Purification of ferredoxin

The purification method for ferredoxin (BL21(DE3)(pDS2)) was modified from the previously reported method [11]. The cell extract from BL21(DE3)(pDS2) was applied to a HiPrep QFF(20ml) column equilibrated with 25mM MOPS,100mM NaCl, 5% glycerol and 0.5mM DTT(buffer II). Unbound protein was eluted with six column volumes of buffer II and the bound protein was eluted with a linear gradient of 0.1M to 0.8M NaCl. The ferredoxin fractions were pooled and concentrated in an amicon MWCO 10 kDa filter unit. Ammonium sulphate was added to the protein to a final concentration of 1.5M, incubated for 2hrs and centrifuged for 20min at 20,000g. The supernatant was loaded onto Hiload superdex S-75 column which was pre-equilibrated with buffer II. Active fractions were pooled, concentrated and buffer exchanged with 50mM MES buffer, pH 6.8.

### Purification of oxygenase

Oxygenase purification method was also modified from the previously reported method[11]. The cell extract from the BL21(DE3)(pDS21) was applied onto a HiPrep QFF(20ml) column equilibrated with 50mM bis-Tris, 5% glycerol and 0.5mM DTT (buffer III). Unbound protein was eluted with six column volumes of this buffer and the bound protein was eluted using a linear gradient of 0 to 0.8M KCl. Oxygenase fractions were pooled and concentrated using an amicon MWCO 100 kDa filter. Ammonium sulphate was added to the protein till a final concentration of 1M is obtained, incubated for 2hrs and centrifuged for 20min at 20,000g. The supernatant was loaded onto a Hitrap butyl sepharose HP column that was equilibrated with buffer III containing 1M ammonium sulphate. Unbound protein was eluted with buffer containing ammonium sulphate and the bound protein was eluted using a linear gradient from 1M to 0M ammonium sulphate. Oxygenase fractions were pooled, concentrated and finally loaded onto Hiload superdex S-200 gel filtration column equilibrated with buffer III. A monodispersed peak of the oxygenase was obtained. The protein fractions were concentrated and buffer exchanged with 50mM MES buffer pH 6.8 before storage.

### Crystallization

Initial crystallization screenings were carried out with Nextal classic suite, Nextal classic suite II, Emerald wizard screen I/II, Crystal screen I /II and Hampton PEG ion I/II. Oxygenase (25-38mg/ml) was crystallized using hanging drop vapor diffusion methods at 277K. Drops containing 0.2 μl protein and 0.2 μl of precipitant were equilibrated against 100μl of reservoir solution for screening. Nanodrop crystallization setup was done with the help of MOSQUITO^®^. Ferredoxin (10-32mg/ml) and reductase (15–26 mg/ml) were also crystallized using hanging drop vapor diffusion at 277K in the screening plate.

### Data collection and processing

Data were collected on the beamline ID29 and ID23-2 at the European synchrotron Radiation Facility (ESRF), Grenoble, France. The oxygenase crystals were frozen by using 20% ethylene glycol as a cryoprotectant. Ferredoxin crystals were frozen without any cryoprotectant. The data were indexed, integrated scaled and merged using Mosflm[24]and SCALA[25].

### Structure determination and refinement

The structure of oxygenase of 3-nitrotoluene dioxygenase was solved by molecular replacement using the program MOLREP[26]. The coordinates of native naphthalene dioxygenase (PDB code: 1NDO) which has the sequence identity of nearly 80% to 3NTDO-O_DS2_ was used as a search model[10]. The structure of ferredoxin was solved by molecular replacement using the ferredoxin of naphthalene dioxygenase from *Pseudomonas* sp. NCIB9816-4(PDB code: 2QPZ)[27]with sequence identity of 76% to 3NTDO-F_DS2_. Refinement was carried out using Phenix[28] and model building was performed by Coot[29].

### Site-directed mutagenesis

Site-directed mutagenesis was performed using the pDS21 plasmid as a template by Quik^®^ Change Site-Directed Mutagenesis system according to the manufacturer’s instruction. The whole plasmid was amplified with mutant primers (Table 1) using the kit protocol and after PCR cycles, the parent template was digested with DpnI enzyme at 37°C overnight and transformed in DH5α cells. Oligonucleotides were synthesized from Bioserve, Hyderabad, India. All mutations were confirmed by DNA sequencing by using both forward IspINF (5’-AACCCACCTTCAAGCACTCTGG-3’) and reverse ACR2 (5’-TTAGCGATCAGTTGTCTTGGTGAGTTCGGTG-3’) primers that were carried out at DNA sequencing facility of National Center for Biological Science, TIFR, Bangalore, India. The resulting derivatives of pDS21 were used to transform in DH5α for sequencing and BL21 (DE3) was used for expression and substrate specificity studies.

**Table 1:** Primers used in this study.

Mutation	Mutagenic oligonucleotide^a^
M251L (For)	5’-TGTGGTTACCGGCGTAGTAGTCCCA*A*A*G*TAGGCTCATT-3’
M251L (Rev)	5’-AGTGGAATGAGCCTA*C*T*T*TGGGACTACTACGCCGGTAAC-3’
N258V (For)	5’-ACTACTACGCCGGT*GTG*CACAGCGCTGATCTGGTT-3’
N258V (Rev)	5’-GAACCAGATCAGCGCTGTG*CAC*ACCGGCGTAG-3’
V350F (For)	5’-ACGCGGTTCAGCGC*ACT*TTCGGACCAGGAGGATAC-3’
V350F (Rev)	5’-CTTTCCCACTATCCTCCTGGTCCGAA*AGT*GCGCTGAAC-3’
I204A (For)	5’-GCGGAAAACTTTGTAGGTGA*TGCA*TACCACGTTGGTTGG-3’
I204A (Rev)	5’-CGTGCGTCCAACCAACGTGGTA*TGCA*TCACCTACAA-3’

^a^ Codons are underlined and italicsindicates bases thatwere changed

### Biotransformation of aromatic substrates

Biotransformation reactions were performed in a 250ml flask containing 50ml total reaction mixture. The reaction mixture composition was same as used previously for the enzyme assay[11], which contained reductase, ferredoxin, oxygenase, NADH, ferrous ammonium sulphate and the substrate (250mM). The reaction was initiated by the addition of oxygenase and it was incubated with shaking at 30°C on a rotatory orbital shaker for an hour. After that, the reaction mixture was acidified (pH 5–6) with 2N HCl. Transformation products were extracted with ethylacetate, dried over anhydrous sodium sulphate and concentrated in vacuo. Products from all mononitrotoluene isomers were identified by GC-MS. The chiral product from naphthalene was also characterized by HPLC. The GCMS parameters and HPLC gradients are same as previously reported [11].

### Docking studies

The ligands were built using the sketch module of Avogadro 1.1.0 (http://avogadro.openmolecules.net). Each substrate was minimized using the universal force field (UFF) available in the Avogadro tool[30]. Crystal structure of the oxygenase was used to perform the docking experiments. The pdb file was used as an input structure to add all hydrogen atoms. The hydrogenated protein was then prepared for docking calculations using the autodocktools package. Docking experiments were performed using Autodock 4.2 (http://autodock.scripps.edu) for the substrate. Other parameters were left to their respective default values. Active site residues were kept as the flexible residues. The results were ranked in terms of the affinity energy and the occurrence of the given docked conformation. All the results were compared with different crystal structures of nitrobenzene dioxygenase (NBDO) and naphthalene dioxygenase (NDO). The pocket volume was calculated with the help of CASTp software tool[31]. The docking conformations were also confirmed by Discovery studio 2.5. Seventeen residues that line the active site of oxygenase were used for docking.

## Results and discussion

### Crystallization of 3NTDO components

The ferredoxin crystallized in 0.2M CaCl_2_, 0.1M HEPES pH 7.5 and 28% PEG 400 at 4 to 8°C. These crystallization conditions were optimized further to 0.1 to 0.25 M CaCl_2_, 22–32% PEG400 and 0.1M HEPES (pH 7.1 to 7.7). Typically, brown crystals (0.2X0.1X0.05mm) appeared after 2–3 weeks.

The oxygenase crystallized in 0.2M sodium citrate tribasic and 20% PEG 3350 pH 8.3 condition at 4 to 8°C. This crystallization condition was optimized to 15–25% PEG 3350 at same temperature. Orange hexagonal plate crystals (0.1X0.1X0.05mm) were obtained after 7 days. Extensive crystal screening with needle shaped crystal of reductase of 3NTDO was carried out; however,it did not yield a diffraction quality crystal.

### Molecular structure of ferredoxin and oxygenase components of 3NTDO

**Fig 1:**
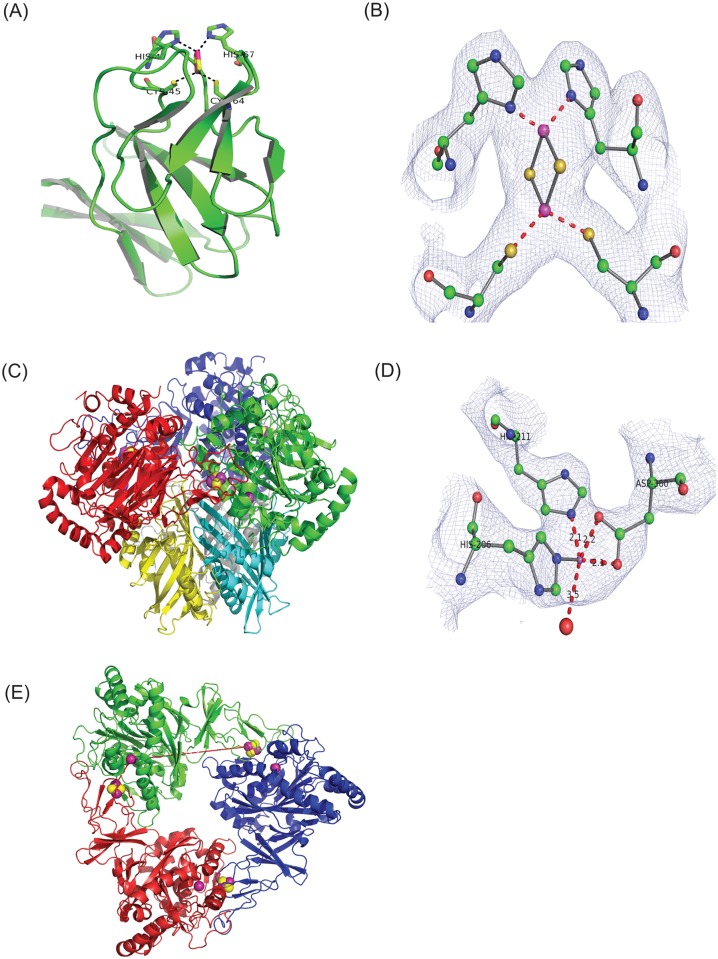
Overall structure of ferredoxin and oxygenase components of 3NTDO. (A) 3NTDO-F_DS2_, the Rieske center and coordinating side chain residues are shown in stick representation. (B) 2Fo-Fc electron density contoured at 1.0 σ level in the Rieske cluster of ferredoxin shown as a ball and stick model and in blue wire mesh. (C) 3NTDO-O_DS2_, the three αsubunits are colored green, blue and red and β subunits are in yellow, grey and cyan. The Rieske center and the catalytic iron are shown in CPK model representation. (D) 2Fo-Fc electron density contoured at 1.5σ level in the vicinity of catalytic iron center with the ligand His206, His211 and Asp360 shown as a ball and stick model. (E) View along the molecular threefold axis of 3NTDO-O_DS2_. The iron, sulphur, nitrogen and water are shown in magenta, yellow, blue and red colors, respectively.

A structure of ferredoxin (3NTDO-F_DS2_) (Fig 1A) was solved to 2.4Å resolution using the molecular replacement method and by using the ferredoxin component of NDO (NDO-F9816-4) (PDB code: 2QPZ)[27] from *Pseudomonas putida* NCIB9816-4 as a search model. The final refined structure has R_work_ = 23% and R_free_ = 26.2% (Table 2). The asymmetric unit contains only one molecule of 3NTDO-F_DS2_. The final model contains a [2Fe-2S] cluster and clear electron density for 3 to 104 residues. Similarly, the data from an orange crystal was used to solve the structure of 3NTDO-O_DS2_ by molecular replacement using the coordinates of NDO (PDB code: 1NDO)[10]. The crystals have one αβ hetero dimer in an asymmetric unit. The structure has been refined to a final R value of 24% (R_free_ = 28%) to 2.9Å resolution. All the residues except the three residues at the C-terminus of the α subunit could be located in the electron density map.

**Table 2:** Data collection and refinement statistics of 3NTDO-F_DS2_ and 3NTDO-O_DS2_.

	Ferredoxin(3NTDO-F_DS2_)	Oxygenase (3NTDO-O_DS2_)
PDB-ID	5BOK	5XBP
Wavelength (Ã…)	0.873	0.976
Resolution range (Ã…)	33.81–2.4 (2.486–2.4)	47.71–2.9 (2.95–2.90)
Space group	I 4 2 2	P 31 2 1
Unit cell	79.53 79.53 108.97 90 9090	178.80 178.80 242.32 90 90 120
Unique reflections	7092 (688)	95735 (4714)
Completeness (%)	99.12 (99.28)	96.2 (96.57)
Mean I/sigma(I)	17.28 (6.27)	7.3 (1.11)
Wilson B-factor	55.6	45.46
R-merge	0.063 (0.799)	0.170 (1.346)
Rpim	0.022 (0.283)	0.113 (1.043)
Multiplicity	8.6 (8.4)	5.2 (3.4)
Mn(I) half set correlation CC(1/2)	0.999 (0.811)	0.984 (0.491)
R-factor	0.2312 (0.5345)	0.2406 (0.3594)
R-free	0.2619 (0.5928)	0.2839 (0.3965)
Number of atoms	816	15339
macromolecules	796	15267
ligands	5	15
water	15	57
Protein residues	102	1908
RMS(bonds)	0.011	0.012
RMS(angles)	1.39	1.56
Ramachandran favored (%)	92	93
Ramachandran outliers (%)	0.99	0.69
Clashscore	12.67	21.44
Average B-factor	51.50	40.10
macromolecules	51.60	40.10
solvent	46.40	39.0

3NTDO-F_DS2_ is an elongated molecule with similar dimensions as those reported for NDO-F_9816-4_ (40Å X 30Å X 20Å). The molecular structure is composed of three antiparallel β-sheets with a Rieske [2Fe-2S] cluster present at the tip of the molecule which is common to other Rieske [2Fe-2S] proteins, such as cytochrome bc_1_[32],b_6_f[33], BphF[34], CarAc[35] and TDO-F[36]. Cys45, His47 (of the β turn of β4 and β5) and Cys64, His67 (of β-hairpin between β6 and β7)coordinates the Rieske [2Fe-2S] center(Fig 1B). The overall sequence identity between 3NTDO-F_DS2_ and NDO-F_9816-4_ is 76% and the structure could be superimposed with an rmsd of 0.43 Å for theCα positions.

3NTDO-O_DS2_ is a mushroom shaped hetero hexamer (α_3_β_3_) protein with a cap and a stem composed of the α_3_ and β_3_ subunits, respectively (Fig 1C). The α-subunit contains Rieske domain (residues 38–158) with a Rieske [2Fe-2S] center and a catalytic domain (residues 1–37, 159–447) that contains a mononuclear iron at the active site (Fig 1D–1E). The completely conserved residues His206, His211 and Asp360 coordinate mononuclear iron[10]. The tertiary and secondary structural elements as well as the essential residues in the electron transfer are conserved between 3NTDO-O and other known oxygenases[10, 36].

#### Proposed electron transfer pathway from ferredoxin to oxygenase

**Fig 2:**
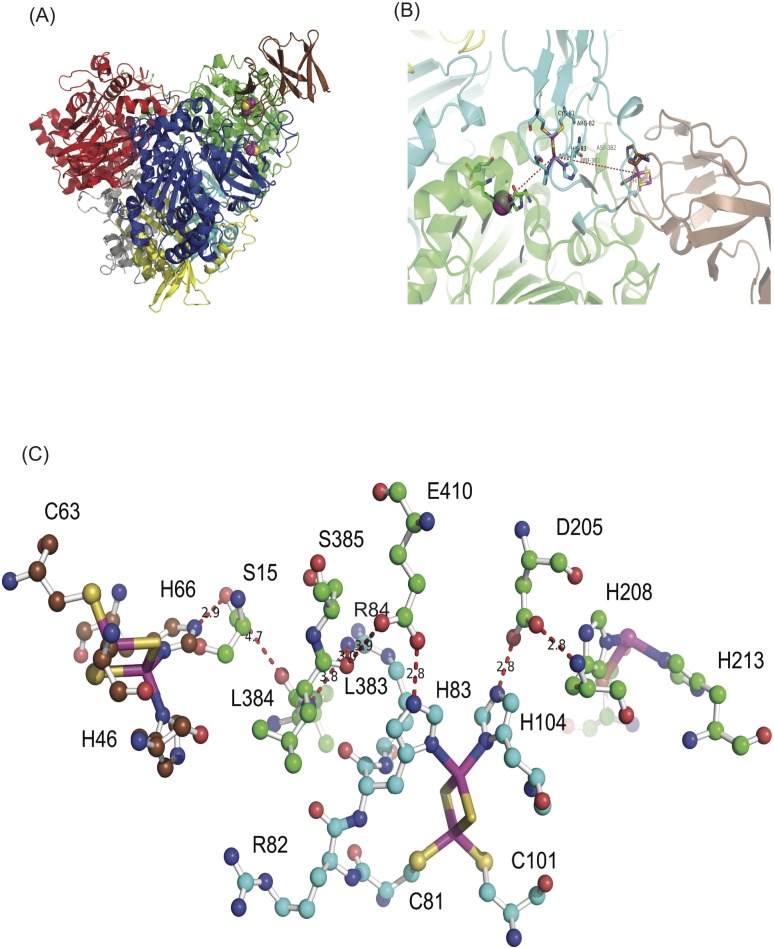
Proposed electron transfer pathway between 3NTDO-F_DS2_ and 3NTDO-O_DS2_. (A) Ferredoxin is shown in brown color at the top of 3NTDO-O_DS2_; Iron and sulphur are shown in magenta and yellow. (B) A line is drawn from Rieske center of 3NTDO-F_DS2_ to the mononuclear iron of 3NTDO-O_DS2_ through the Rieske cluster of 3NTDO-O_DS2_. (C) Proposed electron transfer pathway from Rieske cluster of ferredoxin to active site mononuclear iron of oxygenase of NDO is plotted in ball and stick model, the residues of chain A and chain C of oxygenase are shown in green and cyan color.

The electron transfer pathway from ferredoxin to the α3 type of oxygenase of carbazole 1,9 dioxygenase (CARDO) (PDB code: 2DE5) has been studied previously [37]. For NDO and 3NTDO, the ferredoxin and oxygenase were aligned separately with the oxygenase:ferredoxin complex structure of CARDO. The ferredoxin binding site was proposed by Friemann *et*.*al*[36] and Ashikawa *et*.*al*[37] which was the groove between the two α subunits that represent the cap of mushroom shaped structure (Fig 2A). The average distance between the ligand atoms of the Rieske cluster in oxygenase and ferredoxin of 3NTDO and NDO was 11-12Å (Fig 2B) and it was within the 14Å threshold defining the limit of electron tunneling in proteins[38]. The residues proposed for electron transfer were conserved in both 3NTDO and NDO complex structures. Here, the residues of NDO were used for pathway identification because of the availability of high resolution data. For the proper electron transfer process from the Rieske cluster of ferredoxin to oxygenase, the residues of the two α subunits of the oxygenase are required. In NDO, His66 (NDO-F)-Ser15 (NDO-O)-Leu383-Arg84-Leu384-Glu410-His83 (NDO-O) residues are involved in electron transfer (Fig 2C). Glu410, present at hydrogen bonding distance with the His83 (chain C) reduces the [2Fe-2S] cluster of the oxygenase. In carbazole 1,9-dioxygenase, the proposed pathway has Asp359 that finally transfers electrons from Rieske ferredoxin to the oxygenase[37]. In the naphthalene dioxygenase family, Glu410 plays the role of Asp359 of CARDO system. These sequences of residues are conserved in 3NTDO, NBDO, 2NTDO and DNTDO. During the reaction, the electron flows from Rieske cluster of oxygenase to active site mononuclear iron through Asp205, which is already established [39]. This proposed pathway requires a water molecule [between these pair of residues Ser15 (chain A): Leu383 (chain A); Leu383 (chain A):Arg84 (chain C) and Leu 384 (chain A): Glu410 (chain A)] for electron transfer that is not seen in the modeled structure.

### Substrate specificity of 3NTDO mutants with mononitrotoluenes

**Fig 3:**
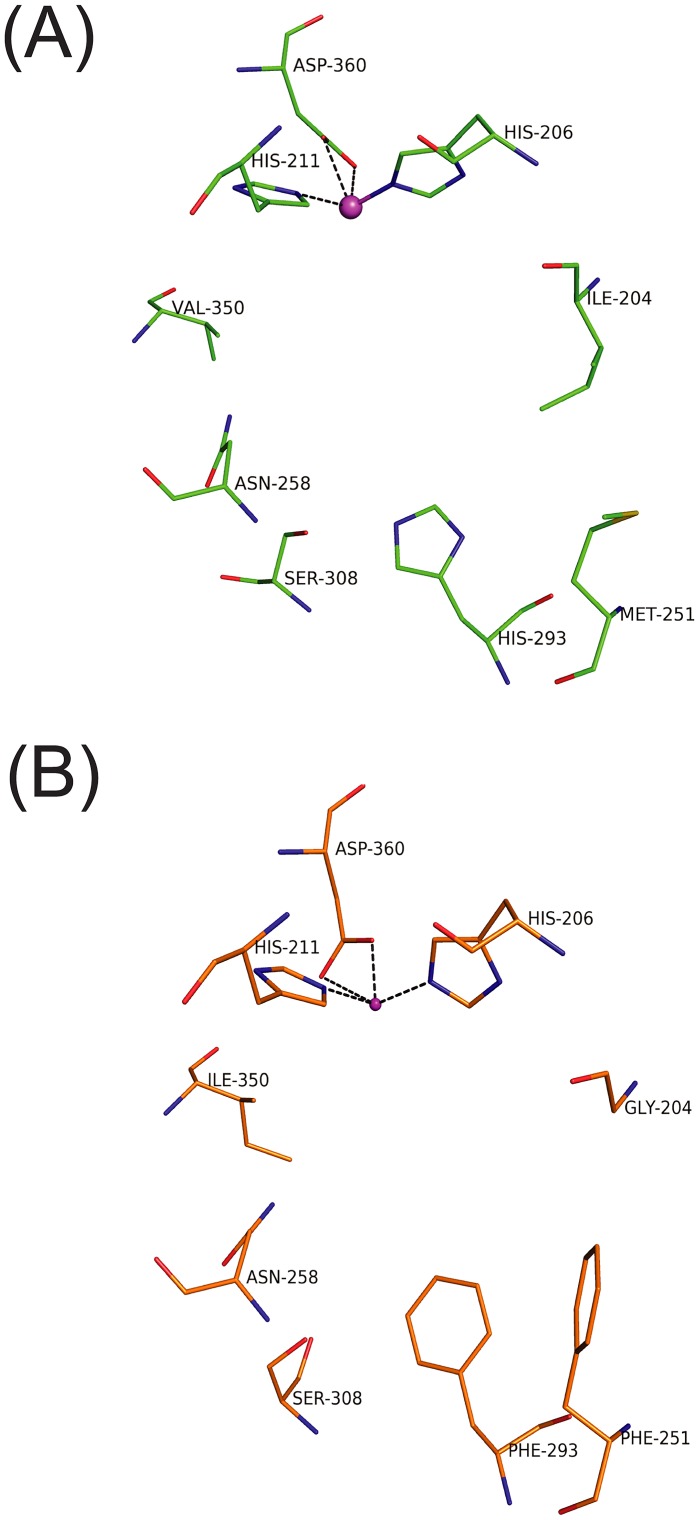
Comparison of active site residues of 3NTDO-O_DS2_ and NBDO. The residues responsible for regioselectivity of 3NTDO-O_DS2_ (A) and NBDO (B) are shown in green and orange stick model, respectively. The mononuclear iron center (large magenta sphere) is coordinated with two histidine and one aspartic residue.

Four residues at the active site in the α subunit of 3NTDO were selected for mutagenesis and were modified based on the sequence of NDO. Ile204, Met251, Asn258 and Val350 which line the pocket of the active site of 3NTDO, were mutated to corresponding residues of NDO (Ala206, Leu253, Val260 and Phe352). To check the expression of the recombinant proteins, crude proteins were run on a 12% SDS-PAGE which showed similar levels of expression. The active site residues of NBDO and 3NTDO are shown in Fig 3.

**Fig 4:**
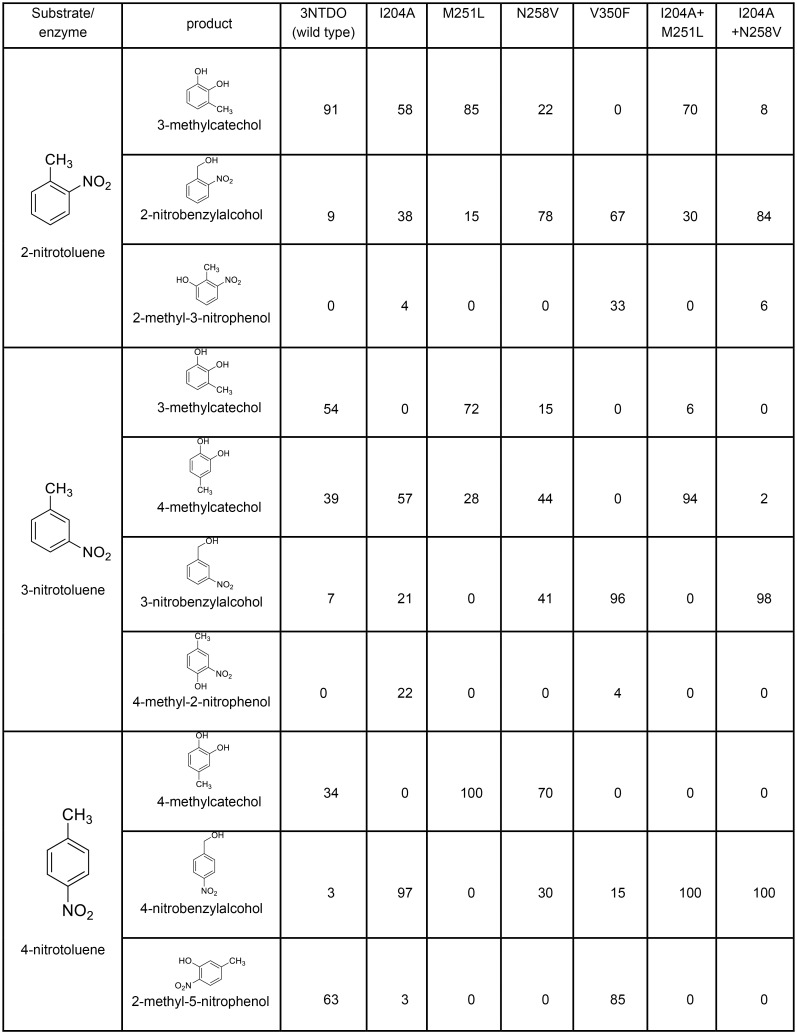
Oxidation products ratios^b^ (%) formed by 3NTDO and its variants with mononitrotoluenes. ^b^Product ratios were determined from by theintegration of Gas chromatography mass spectrometry (GC-MS) total ion current chromatogram. Results reported are the average of at least three independent experiments, and standard deviations were less than 7%.

The ability of the mutant enzymes to oxidize 2-nitrotoluene (2NT), 3-nitrotoluene (3NT) and 4-nitrotoluene (4NT) were also checked and the results are presented in Fig 4.

Met251 is present only in 3NTDO, while NDO and NBDO have Leu253 and Phe251, respectively. M251L mutation had little effect on the product ratios formed from 2NT and 3NT (Fig 4). However, a significant change was found in the formation of 4-methylcatechol (4MC) as the sole product from 4NT which was not a major product with the wild type 3NTDO. The major product of wild type 3NTDO was 5-methyl-2-nitrophenol (63%) from 4NT and 5-methyl-2-nitophenol was unique among all the nitoaromatic dioxygenase systems as there is no report available for the formation of this product from 4NT either from wild type or mutated NDO[22], NBDO[17], 2NTDO[18] and DNTDO[15].

Similarly, I204A mutation produced aromatic ring monohydroxylated 4-methyl-2-nitrophenol (22%) from 3NT and 2-methyl-3-nitrophenol (4%) from 2NT (Fig 4) which were not the products either from the wild type 3NTDO or wild and mutants of NBDO[17], 2NTDO [18], NDO [22] and DNTDO[15]. On comparing the formation of methylcatechols from all isomers of mononitrotoluenes, it was found that nitrite release from aromatic systems also decreased. The I204A mutation gave 58% 3MC from 2NT and 57% 4MC from 3NT and no 3MC from 3NT. In 4NT, no catechol formation was detected and it catalyzed the production of 4-nitrobenzylalchohol (4NBA) (97%) as the major product which was only 3% in the wild type 3NTDO. This position was found to be important in dinitrotoluene dioxygenase (DNTDO) from strain *Burkholderia* sp. DNT[15]. I204L and I204Y variants of DNTDO have been reported to biotransform other dinitrotoluene compounds such as 2,3-dinitrotoluene and 2,5-dinitrotoluene, 4NT to 4MC (major product) and 2NT to 2-nitrobenzylalcohol (2NBA) [12].

Asn258 of NBDO and 2NTDO were proposed to be important residues in positioning the nitro substrate for the oxidation. Asn258 was found to form a hydrogen bond with the nitro group of nitrobenzene and 3-nitrotoluene in the substrate bound crystal structure of NBDO[13]. Replacement of Asn-258 with Val in 3NTDO resulted in decreased specificity for attack at the nitro group of 2NT and 3NT. As a result 2-nitrobenzyl alcohol (2NBA) and 3-nitrobenzyl alcohol (3NBA) were formed as the dominant products from 2NT and 3NT, respectively while from 4NT, this variant did not show any change and gave 4MC as a major product instead of 4-nitrobenzylalcohol (4NBA) (Fig 4). On comparing these results with 2NTDO and NBDO mutagenesis studies, it was observed that N258V variant of 3NTDO behaved like N258V variant of 2NTDO and produces 82% 4MC from 4NT [18] while N258V variant of NBDO produces 99% 4NBA from 4NT [17]. The ratio of 4MC from 3NT formed from N258V variant of 3NTDO (41%) did not change with wild type of 3NTDO (39%)while in 2NTDO, N258V mutation produces 80% of 3NBA. This indicates that the presence of an aspargine at 258 position is important only for the formation of 3MC from 2NT and 3NT and has no effect in the formation of 4MC from 3NT and 4NT. The wild type DNTDO from *Burkholderia* sp. DNT has Val at 258, produces4MC as a dominant product from 4NT [15]. It is clear from the above observations, that N258 in 3NTDO is not the only responsible residue to remove nitrite from all isomers of mononitrotoluene. In NBDO and 2NTDO, this variant is unable to detach nitrite from 2NT and 3NT significantly[18]. In I204A/M251L variant of 3NTDO, 4MC was observed as a dominant product (96%) over 3MC from 3NT. Here, it behaved like wild type NBDO in which 4MC formation takes place from 3NT substrate. I204A/N258V mutation gave nitrobenzyl alcohol as a major product from all isomers of mononitrotoluene. These results suggest that, for nitrite release from mononitrotoluenes both Ile204 and Asn258 are equally important and their role is the proper anchoring of the substrate in 3NTDO.

The V350F variant produced 2NBA and 3NBA as dominant products from 2NT and 3NT, respectively. This substitution in 2NTDO produces trace amounts of methylcatechols from the respective mononitrotoluenes whereas in our case only methylnitrophenol was identified from all isomers of mononitrotoluene (Fig 4). Similarly in NBDO and 2NTDO, this mutation lost its ability to release nitrite from the substrates while it retained the ability to oxidize naphthalene to (1*R*, 2*S*)-*cis*-1,2-dihydro-1,2-dihydroxynaphthalene like the wild type 3NTDO. The introduction of larger residue at this position most likely reduces the volume of the active site pocket and interferes with the correct orientation of nitrotoluene that is necessary to yield the corresponding catechols[18]. In order to understand the structural basis of the formation of other nitrophenol products, the volume of the active site pocket (wild type) of 3NTDO, NBDO and NDO were calculated from the respective crystal structures by using CASTp web server[31]. The pocket size was found to be 445Å^3^(3NTDO), 295Å^3^(NBDO) and 693Å^3^ (NDO) respectively. This suggests that 3NTDO has a bigger pocket size than NBDO and this could explain the formation of other nitrophenol products–by facilitating binding of the larger substrate. The V350T variant in NDO from *Ralstonia* U2, removes the nitrite from 2,3- and 2,6-dinitrotoluene, but not from 2,4-dinitrotoluene[21]. It is the pocket build by hydrophobic residues in the active site that orient the substrate in the right direction for catalysis. The multiple mutations carried out in NDO from *Pseudomonas putida* NCIB 9816–4 were also reported to be inactive in nitrite release from mononitrotoluenes[22]. This could be because of large pocket size of NDO which was unable to lock the substrate in the catalytically relevant orientation. The crystal structure of NDO with 3NT gave two orientations in which one orientation could explain the formation of benzyl alcohol[40]. When we compared the residues which were mutated for making hybrids of NBDO in NDO, we observed that some residues were less hydrophobic and these could increase the pocket size which do not favour for proper anchoring and orientation of the substrates like mononitrotoluene. The residues which were present at the active site of DNTDO also seemed to give a smaller pocket than NDO. Thus it might be possible to engineer the NDO and DNTDO for releasing nitrite from mononitrotoluene by tuning the pocket size.

**Fig 5:**
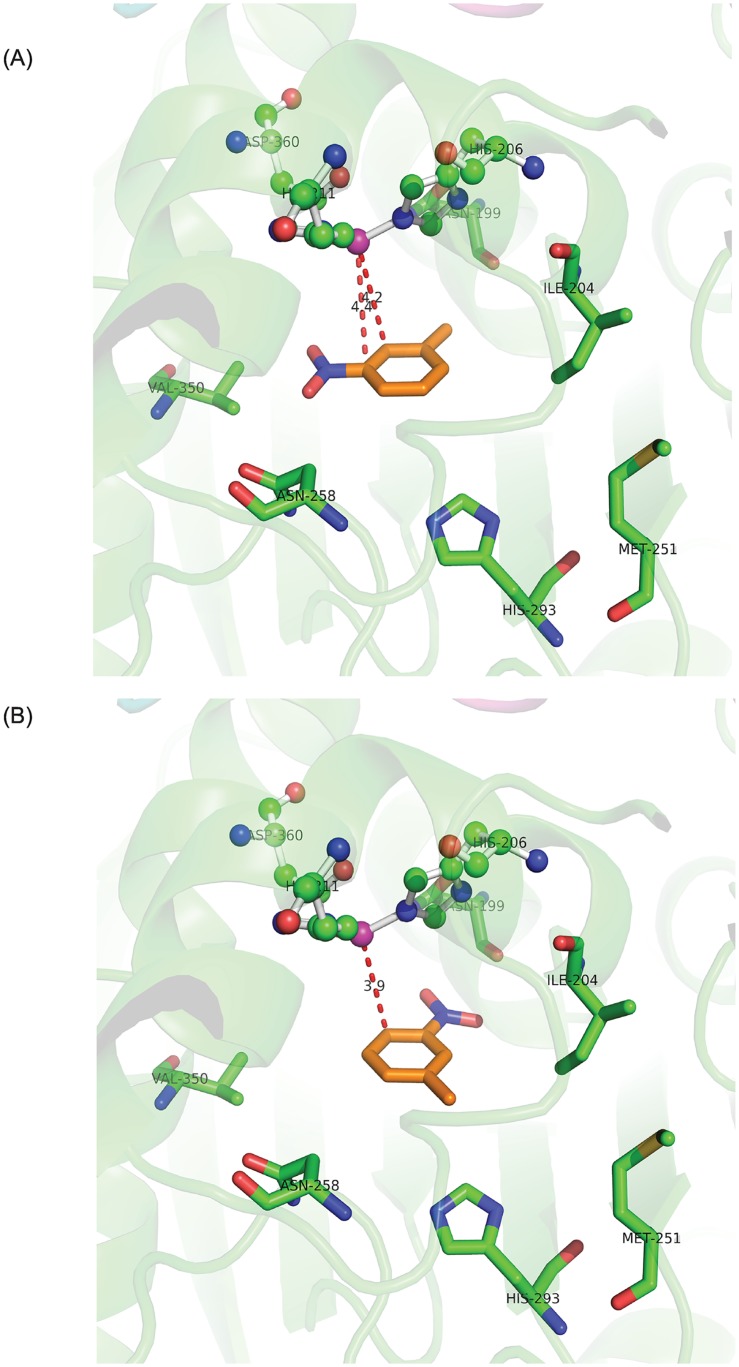
Docked conformations of 3-nitrotoluene in the active site of 3NTDO-O_DS2_. (A) 3-methylcatechol product orientation (B) 4-methyl-2-nitrophenol orientation. The distance between mononuclear iron and carbon atoms of benzene ring is shown in red dotted lines. The ligands His208, His211, Asp360 and mononuclear iron (magenta) are shown in ball and stick model. The active site residues are shown in stick and 3-nitrotoluene is in orange color.

We also docked the substrates in the active sites using Accelrys Discovery studio 2.5 and AutoDock Vina. The most stable conformation was observed for 3-methylcatechol (Fig 5A) which agreed with the biotransformation results in which the major product was 3MC from 3NT (Fig 4).

Docking score was calculated as -2.74kcal/mol and binding affinity was -6.9kcal/mol. The distance between mononuclear iron and carbon atoms (C2 and C3) adjacent to nitro group were 4.2 Å and 4.4 Å, respectively. It was observed that on docking, the methyl group of the substrate interacts with Val207 and Ile204 residues of the active site at the entrance of the pocket. The least stable conformation was for 4-methyl-2-nitrophenol product orientation (-5.8Kcal/mol)(Fig 5B) which was the product for 3NTDO variants and not for the wild type protein. The binding affinities for 4-methylcatechol and 3-nitrobenzylalcohol were -6.5kcal/mol and -6.2kcal/mol, respectively.

Our results suggest that the substrate specificity of 3NTDO is quite different from NBDO and 2NTDO. While the N258V mutation in other dioxygenases affects nitrite release prominently from mononitrotoluene substrates, in 3NTDO; the double mutation Ile204Ala/Asn258Val changes the regioselectivity of all mononitrotoluene isomers. Ile204 residue is found to be equally important for proper anchoring the substrates and for removing nitrite. The residue Val350 is also important as it changes the regioselectivity. The proposed electron pathway from Rieske center of the ferredoxin to that of the oxygenase active site can be further checked to identify residues responsible for the electron transfer. Efforts to improve the regioselectivity with the range of substrates through protein engineering and complex crystallization of ferredoxin and oxygenase are underway.
